# Phenomenological and Thermodynamic Model of Gas Exchanges in the Placenta during Pregnancy: A Case Study of Intoxication of Carbon Monoxide

**DOI:** 10.3390/ijerph16214138

**Published:** 2019-10-27

**Authors:** Juliana Rangel Cenzi, Cyro Albuquerque, Carlos Eduardo Keutenedjian Mady

**Affiliations:** 1 School of Mechanical Engineering, University of Campinas, Mendeleyev St., 200 - Cidade Universitária, 13083-970 Campinas, Brazil; juliana.cenzi@gmail.com; 2 Department of Mechanical Engineering, Centro Universitário da FEI, 09850-901 São Bernardo do Campo, Brazil; cyroan@fei.edu.br

**Keywords:** exergy analysis, respiratory system, carbon monoxide intoxication, placenta

## Abstract

The present work simulates the transport of oxygen, carbon dioxide, and carbon monoxide between a fetus’s circulatory system and the mother’s. The organ responsible for this exchange is the placenta. Carbon monoxide is a common air pollutant, and it impacts the physiological conditions even in low concentration. The impacts of carbon monoxide are especially dangerous for pregnant women, fetuses, and newborn babies. A model of carbon monoxide transport, from the literature, is modified to simulate a pregnant woman (original model was a male), therefore changing some parameters to express the adjusted respiratory system. It was considered the gas exchange in the placenta, to evaluate the concentration of these different gases in the fetus arterial and venous blood. Three methods of the exergy analysis are implemented for both mother and fetus respiratory systems, aiming at the comparison with the respiratory system of a male adult. The destroyed exergy of the literature did not have the same trend as the models proposed in this article, taking into consideration the hemoglobin reactions. In contrast, the entropy generation associated only with the diffusion transport phenomena was one order of magnitude lower than the other methods. The placenta destroyed exergy rate is significantly higher compared to the irreversibilities of the mother’s respiratory system. One possible explanation is the fact that the placenta has other physiological functions than gas transportation.

## 1. Introduction

The application of the Second Law of Thermodynamics to living beings has its initial applications from the middle of the past century with two symbolical works proposed by Schrödinger [1] and Prigogine and Wiane [2]. These works open a new field of application of the Second Law of Thermodynamics, nowadays usually applied by means of the Exergy Analysis. As proposed by Oliveira [3], one of the most interesting applications of the Second Law is understanding the functioning of living beings and use the physical property entropy generation (or destroyed exergy) as an indicator or diagnosis of pathology. This assessment tool was applied to a cancerous cell [4], neuron under hypoxia [5], human lungs [6,7], human heart [8,9,10], athletes under different training level [11,12], hypothermia as a therapy [13], orientated healing of burned patients [14] and, the most common application, the analysis of thermal comfort conditions [15,16,17] (comparison of the comfort indexes with destroyed exergy and exergy efficiency) systematized in [18]. Furthermore, Ozilgen [19] carried out an extensive review of this area, pointing out that this field has promising applications in several areas of interaction of Thermodynamics with living beings’ behavior.

Regarding the pollutant carbon monoxide (CO), it is considered to be a harmful gas since it bounds to hemoglobin, decreasing the blood capacity to deliver oxygen to tissues [20]. Pregnant women can be poisoned by work-related sources like some industrial process (hot furnaces, gas refineries), or household sources like defective water heaters, or even passing through a tunnel in an urban environment. Smoking or being a passive smoker can also cause damage: a smoke-filled room has a carbon monoxide concentration as high as 100 ppm. This concentration is enough to cause a chronic poisoning, characterized by carboxyhemoglobin (HbCO) in the 5–25% range.

The bind between O${}_{2}$ and hemoglobin (Hb) forms oxyhemoglobin ($HB_{O_{2}}$). Each hemoglobin can carry four molecules of O${}_{2}$. The easiness of the reaction increases when the first oxygenation occurs. The gas CO is toxic because it has an affinity with the hemoglobin 200–300 times greater than O${}_{2}$. Hence, even relatively small concentrations of CO inspired can reduce the delivered oxygen to the tissues, causing hypoxia. Therefore, the toxicity of CO is related to the increase of carboxyhemoglobin (reaction of carbon monoxide with hemoglobin). The usual treatment of CO poisoning consists of reducing the inspired CO to zero and increasing the oxygen inspired, thus generating an increment in the gradients of these gases in the alveolar ventilation and increasing O${}_{2}$ partial pressure in the blood [7,21].

The intoxication symptoms usually are headaches, sensations of weakness, dizziness, sleepiness, impaired physical performance, visual difficulties, palpitations, nausea, and vomiting [22]. Effects in fetus depend on the pregnancy stage in which the poisoning occurs. During the embryonic stage, it can cause skeletal and neurological effects [22]. Later in pregnancy, it is responsible for anoxic encephalopathy, which occurs when there is not enough O${}_{2}$ delivered to the fetus’ brain. During gestation, oxygen and nutrients are provided to embryo through the placenta, which uses several transport mechanisms to accomplish this task: simple or facilitated diffusion, active transport, pinocytosis and phagocytosis. Transport of O${}_{2}$ and CO${}_{2}$ occurs mainly by simple diffusion [23].

Herein, O${}_{2}$, CO${}_{2}$ and CO transport are modeled to occur as a result of simple diffusion of the number of gases dissolved in maternal blood. However, some authors suggest that, for carbon monoxide, this mechanism could happen due to facilitated diffusion [22]. Therefore, this work aims at modeling and expanding the phenomenological behavior of a healthy male human lung subjected to the inhalation of O${}_{2}$, CO${}_{2}$, and CO, first proposed by Albuquerque Neto et al. [20]. Moreover, based on the exergy method proposed by Henriques et al. [6] and Cenzi et al. [7], the objective is to expand the phenomenological and exergy model to a pregnant woman subjected to conditions usually found in urban ecosystems and during stress periods such as passive smoking or prolonged exposure to a tunnel environment. The distinguishing feature of this article is the application of these conditions to a pregnant woman, with a phenomenological of the mother [20] and fetus (proposed in the present article).

## 2. Methods

### 2.1. Maternal Respiratory System

The maternal respiratory system is considered to work independently of the placenta. In other words, pregnancy does not affect the gas exchanges in the lungs, although there exist modifications in some parameters of the respiration, such as the volumetric ventilation [21,24,25,26]. Considering this fact, it was possible to use the model proposed by Albuquerque Neto et al. [20]. The work proposed a multicompartment model for the respiratory system. It allowed calculating partial pressures and saturation levels for CO${}_{2}$, CO, and O${}_{2}$ for the arterial and venous blood. During pregnancy, 2,3-Diphosphoglycerate concentration increases [22], shifting the oxygen dissociation curve to the right. This shift in concentration was not taken into account in this work since the original dissociation curve implemented by Albuquerque Neto et al. [20] was used and took this fact into account.

**Figure 1:**
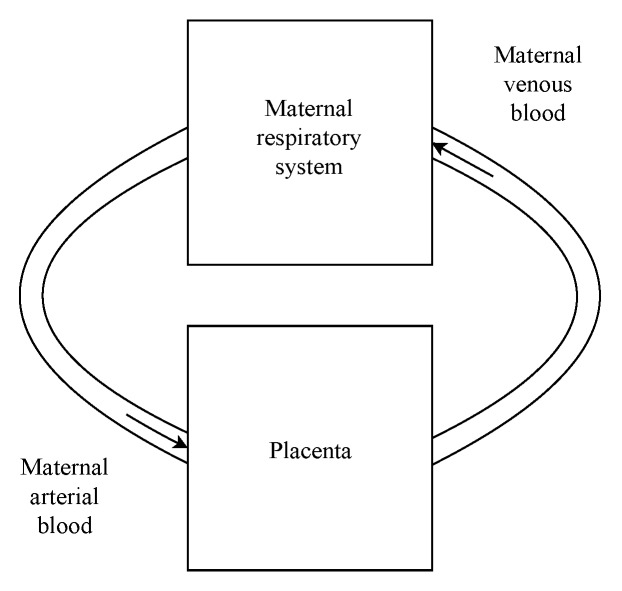
Gas exchange model for pregnancy indicating the mother respiratory system obtained in [20] and the gas exchanges in the placenta proposed in the present analysis.

Maternal arterial blood leaves the respiratory system (hence, lungs), from where it is pumped by the heart [10] (left systolic ejection). From this point, it goes to the placenta, where gases and nutrients exchanges occur, leaving it as venous blood. Eventually, it flows through the heart, leaving the systemic circulation and entering the pulmonary circulation. Ultimately, it returns to the lungs, where it is oxygenated, considering that the work performed by the heart is not evaluated in the present analysis (this analysis was performed elsewhere [10]). Therefore, it is omitted from Figure 1, in which the gas exchange model for pregnancy indicating the mother respiratory system obtained (and modified) in [20] and the gas exchanges in the placenta proposed in the present analysis are sketched.

The input data applied to the model are summarized in Table 1. They were used to represent physiological pregnancy conditions with more precision. These parameters were obtained from Albuquerque et al. [20].

### 2.2. Placenta Gases Exchange

The placenta is an organ that is responsible for performing the exchange between mother and fetus. It plays the role of the lungs, intestines, and kidneys of the fetus during the pregnancy. It has a high metabolism level, as is indicated by its high O${}_{2}$ and glucose consumption. This organ has two independent circulatory systems: the maternal and the fetus. Arterial blood from the uterine arteries enters the placenta, constituting a lake of arterialized blood. It is in this lake that exchanges take place. Carbon dioxide and waste products come from the umbilical arteries and are exchanged by passive diffusion to the maternal blood. Oxygen and nutrients diffuse from the maternal blood to the fetus [27]. The gas exchange process in the placenta can be modeled as a concurrent process. Table 2 lists important parameters of umbilical blood flow.

Similarly to the work executed by Sharan et al. [29], the placenta was modeled as two compartments: one for the maternal blood flow and other representing the fetal blood flow. The pressure of any gases inside it is considered constant and equal to the pressure at which it leaves the correspondent compartment. Maternal arterial blood enters at a given oxygen pressure ($P_{mar}$), 90 mmHg, and leaves at venous concentration, 40 mmHg, $P_{mve}$. Fetal arterial blood enters with an oxygen pressure ($P_{far}$) of 25 mmHg and leaves as venous blood ($P_{fve}$) at 40 mmHg. Umbilical veins receive these names, because, even though arterial blood is poor in oxygen, it leaves the fetus heart. The rate of of gas volumetric diffusion ($\dot{V}$) for a given gas is supplied by Equation (1) [20,29]:

(1) $$\dot{V}=D_{L}\left(P_{m,ve}-P_{f,ve}\right)$$

In this equation, $D_{L}$ (mL·min${}^{-1}$·kg${}^{-1}$) is a diffusion coefficient and $P_{i}$ (mmHg) is the gas partial pressure in the compartment. The mass flow rate (kg·s${}^{-1}$) at which the blood gains a gas is indicated in Equation (2).

(2) $${\dot{m}}_{diffusion}=\dot{V}\left(C_{ar}-C_{ve}\right)$$

where $\dot{V}$ is the blood flow rate (m${}^{3}$·s${}^{-1}$) and *C* is the gas total concentration (kg·m${}^{-3}$) in blood and can be evaluated by Equation (3) [20,29].

(3) $$C=\alpha_{b}P+N\left[Hb\right]S\left(P\right)$$

where *S(P)* is a function to represent the oxygen dissociation curve, α is the solubility coefficient of the gas in the blood (ml${}_{O_{2}}$·ml${}_{blood}^{-1}$·mmHg${}^{-1}$), [Hb] is the hemoglobin concentration (g/mL) and N is the total carrying capacity of the blood ($\frac{O_{2}\left[ml\right]\left[ml\right]}{blood[ml][g]}$).

To obtain carbon monoxide concentration in fetal venous blood, in steady-state conditions, Equation (4) was solved numerically using the bisection method until the pressure change within an error as in Equation (5). It is important to note that Equation (4) is the combination of Equations (1) and (2) and that the carbon monoxide pressure in the arterial fetal blood compartment is the same as the venous one for the last iteration (Equation (6)).

(4) $$D_{L}\left(P_{m,ve}-P_{f,ve}^{n}\right)={\dot{V}}_{b}(C_{f,ve}\left(P_{f,ve}^{n}\right)-C_{f,a}\left(P_{f,ve}^{\left(n-1\right)}\right)$$

(5) $$|\left(P_{f,ve}^{n}-P_{f,ve}^{\left(n-1\right)}\right)/\left(P_{f,ve}^{n}\right)|<\epsilon$$

(6) $$P_{f,ar}^{n}=P_{f,ve}^{\left(n-1\right)}$$

### 2.3. Exergy Analysis

The exergy analysis for a control volume (CV) can be obtained from Equation (7). The term *b* is the exergy of a stream, which is $b=h-h_{0}-T_{0}\left(s-s_{0}\right)$. This equation may be considered as the degradation of the quality of the energy [3] for a given reference environment: $P_{0}=1$ atm and $T_{0}=25$ °C.

(7) $$\frac{dB_{CV}}{dt}=\sum {\dot{m}}_{i}b_{i}\sum {\dot{m}}_{o}b_{o}+{\dot{Q}}_{CV}\left(1-\frac{T_{0}}{T_{CV}}\right)-{\dot{W}}_{CV}$$

In this equation, $\frac{dB_{CV}}{dt}$ is the exergy variation of the control volume, $\sum {\dot{m}}_{k}b_{k}$ is the exergy rate carried in or out of the control volume by a stream, and ${\dot{Q}}_{CV}\left(1-\frac{T_{0}}{T_{CV}}\right)$ is the exergy transfer rate associated with a heat at $T_{CV}$ to the reference $T_{0}$.

Three different approaches to the exergy analysis were performed at each system. These methods were obtained in Cenzi et al. [7]. The first method considers that the gases contained in the blood are carried dissolved with its partial pressure [6]. The second one takes into account that oxygen and carbon monoxide are also carried bounded to the hemoglobin [7]. Finally, the Second Law was applied to the system to evaluate the intrinsic entropy generation due to diffusion between regions with finite pressure gradients. The primary purpose is to assess how the intoxication of carbon monoxide may modify the exergy behavior of the mother lungs, which should be similar to the results obtained in [7]. Moreover, there is an objective to understand the fetus’s behavior for a pregnant woman since the mother’s blood is drastically modified in environments that have abnormal concentrations of CO. These scenarios occur in several locations in the urban ecosystem.

#### 2.3.1. Respiratory Exergy and Entropy Generation Analyzes

The different analysis are described for the equations shown bellow. As mentioned, Model 1 was based on the analysis proposed by Henriques et al. [6] where all gases are considered dissolved in blood with its own partial pressure. Equation (8) indicates a exergy of a stream (b in kJ·kg${}^{-1}$) with and the simplification to an ideal gas *i*, with constant specific heat $c_{p_{i}}$[3]. In Equation (8), *h* stands for the specific enthalpy (kJ·kg${}^{-1}$), *s* stands for the specific entropy (kJ·kg${}^{-1}$K${}^{-1}$), and $R_{i}$ is the ideal gas constant in mass basis for the gas *i*. The terms $T_{0}$ and $P_{0}$ are the temperature and pressure in the reference state [30,31].

(8) $$b_{i}=h_{i}-h_{0}-T_{0}\left(s_{i}-s_{0}\right)=c_{p_{i}}\left(T_{i}-T_{0}-T_{0}ln\frac{T_{i}}{T_{0}}\right)+R_{i}T_{0}ln\frac{P_{i}}{P_{0}}$$

Model 2 was based on a modification proposed by Cenzi et al. [7] to insert in the terms related to the exergy release (or absorption) associated with the bonds of O${}_{2}$ and CO with hemoglobin ($\Delta B_{Hb,i}=-\Delta G_{Hb,i}$). The CO${}_{2}$ is mostly transported as bicarbonate ion in the blood, according to the reaction of dissociation. Eventually, it was possible to evaluate the exergy variation between the venous blood and the arterial blood ($\Delta B_{blood,reaction}$), according to Equation (9). A complete description of the calculus of those terms are systematized in [7,32,33]

(9) $$\Delta B_{blood,reaction}=\Delta B_{CO_{2}}+\Delta B_{HB,CO}+\Delta B_{HB,O_{2}}$$

Finally, Model 3, also proposed by Cenzi et al. [7], evaluates only the entropy generation related to mass transfer by diffusion (therefore not considering other metabolic effects, heat and mass transfer with the rest of the body).

- **Model 1**: O${}_{2}$, CO${}_{2}$ and CO dissolved in blood as ideal gases.
  Destroyed exergy rate is obtained by is given by Equation (10), which is an adaptation of the general case (Equation (7)) [7].
  (10) $${\dot{B}}_{d_{lung}}={\dot{B}}_{M_{lung}}+{\dot{B}}_{bl_{ve}}+{\dot{B}}_{air_{ins}}+{\dot{W}}_{resp}-{\dot{B}}_{bl_{ar}}-{\dot{B}}_{air_{exp}}-{\dot{Q}}_{M,lung}\left(1-T_{o}/T_{lung}\right)$$
  Because each stream has its exergy specified, only in this model, it is possible to evaluate an exergy efficiency [7], as in Equation (11).
  (11) $$\eta =\frac{{\dot{B}}_{bl_{ar}}+{\dot{B}}_{air_{exp}}+{\dot{Q}}_{M,lung}\left(1-T_{o}/T_{lung}\right)}{{\dot{B}}_{M,lung}+{\dot{W}}_{resp}+{\dot{B}}_{bl_{ve}}+{\dot{B}}_{air_{ins}}}$$
  In these equations, ${\dot{B}}_{bl}$ refers to exergy flow rate associated with the blood. The Indices ar and ve refer to arterial and venous, respectively. ${\dot{B}}_{air}$ is the exergy flow rate associated with air, exp denotes expired air and ins inspired air. ${\dot{Q}}_{M,lung}$ is heat transfer from the lungs to the tissues surrounding it. The temperature $T_{0}$ is the reference temperature and $T_{lung}$ is lung’s temperature. The term ${\dot{B}}_{M,lung}$ is the metabolic exergy reaction rate associated with lungs’ metabolism, evaluated in [6,34]. ${\dot{W}}_{resp}$ is the power done by the intercostal muscles into the respiratory system [35].
- **Model 2**: Considering the bound of O${}_{2}$ and CO with the hemoglobin.
  In this situation, only the destroyed exergy rate is calculated, which is justified in [7], where the methodology used to evaluate the bound to hemoglobin only allow to determine an exergy difference after and before the bounding ($\Delta {\dot{B}}_{blood,reaction}$) between the arterial and venous blood. These exergy variations were obtained from the Gibbs variation of the reactions, better described in [7].
  (12) $${\dot{B}}_{d_{lung}}={\dot{B}}_{M_{lung}}+{\dot{B}}_{air_{ins}}+{\dot{W}}_{resp}-{\dot{B}}_{air_{exp}}-{\dot{Q}}_{M,lung}\left(1-T_{o}/T_{lung}\right)-\Delta {\dot{B}}_{blood,reaction}$$
- **Model 3**: Entropy generation caused by spontaneous diffusion.
  This method does not take into consideration the bounds of the gases with hemoglobin since it isolated the effects of the transport mechanism. Therefore, it is possible to calculate the irreversibilities (or entropy generation $\dot{\sigma }$ solely on the mass diffusion. The destroyed exergy rate for this system is given by Equation (13):
  (13) $${\dot{B}}_{d_{lun}}=-T_{0}\dot{\sigma }$$
  where $\dot{\sigma }$ is:
  (14) $$\Delta {\dot{S}}_{O_{2}}=\left({\dot{m}}_{O_{2_{ar}}}-{\dot{m}}_{O_{2_{ve}}}\right)R_{O_{2}}\ln\frac{P_{O_{2_{ar}}}}{P_{O_{2_{ex}}}}$$
  (15) $$\Delta {\dot{S}}_{CO}=\left({\dot{m}}_{CO_{ar}}-{\dot{m}}_{CO_{ve}}\right)R_{CO}\ln\frac{P_{CO_{ar}}}{P_{CO_{ex}}}$$
  (16) $$\Delta {\dot{S}}_{CO_{2}}=\left({\dot{m}}_{CO_{2_{ar}}}-{\dot{m}}_{CO_{2_{ve}}}\right)R_{CO_{2}}\ln\frac{P_{CO_{2_{ar}}}}{P_{CO_{2_{ex}}}}$$
  (17) $$\dot{\sigma }=\Delta {\dot{S}}_{O_{2}}+\Delta {\dot{S}}_{CO}+\Delta {\dot{S}}_{CO_{2}}$$
  where for each gas ${\dot{m}}_{ar}$ is the mass flow rate of the gas in arterial blood flow and ${\dot{m}}_{ve}$ in the venous blood. $P_{g_{ex}}$ is the pressure of the gas in the expired air, while $P_{g_{ar}}$ is the pressure in the arterial blood.

#### 2.3.2. Respiratory Exergy and Entropy Generation Analyzes

**Figure 2:**
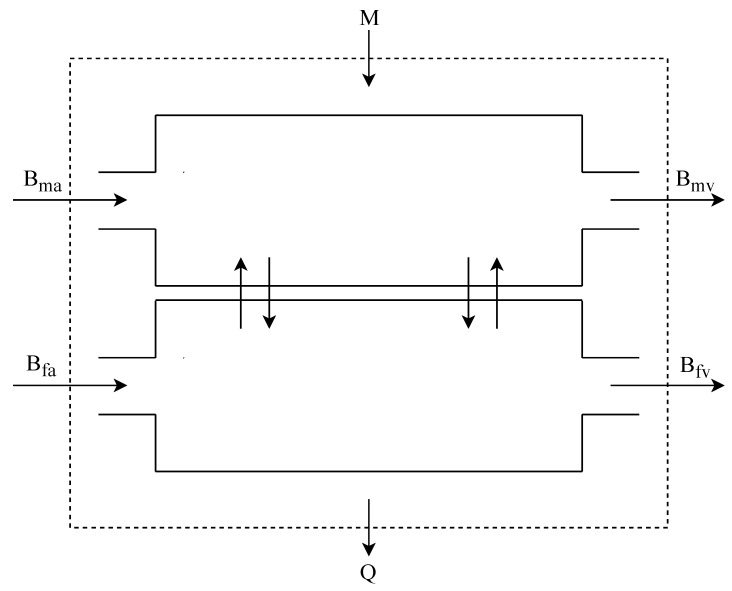
Placenta with the stream of the mother and fetus arterial and venus blood.

The analysis used for the maternal respiratory system is adapted for the placenta. Figure 2 shows how the placenta is modeled. Therefore, as a mass exchanger with its own metabolism (which is expected from an organ). It is important to bear in mind that the fetus tissue forms the placenta, and, therefore, is an organ from the son.

- **Model 1**: O${}_{2}$, CO${}_{2}$ and CO dissolved in blood as ideal gases.
  In the following equations, *m* refers to maternal blood, while *f* to fetal. In addition, the subscript ve refers to venous blood and ar to arterial. ${\dot{B}}_{M}$ is the exergy rate associated with the metabolism and $\dot{Q}$ is heat transferred from the placenta to it surroundings. $T_{0}$ is the reference temperature and $T_{c}$ is the placenta temperature. The efficiency, based on the discussions carried out in [3,30], is defined as:
  (18) $$\eta =\frac{{\dot{B}}_{ve,m}+{\dot{B}}_{ve,f}+{\dot{Q}}_{m}\left(1-T_{0}/T_{c}\right)}{{\dot{B}}_{M}+{\dot{B}}_{ar,m}+{\dot{B}}_{ar,f}}$$
  Destroyed exergy rate is given by Equation (19):
  (19) $${\dot{B}}_{d}={\dot{B}}_{ar,m}+{\dot{B}}_{ar,m}+{\dot{B}}_{M}-{\dot{B}}_{ve,m}-{\dot{B}}_{ve,f}-\dot{Q}\left(1-T_{M}/T_{c}\right)$$
- **Model 2**: Considering the bound of O${}_{2}$ and CO with the hemoglobin.
  To apply this model to the placenta, it was necessary to evaluate the Gibbs free energy related to the gases binding to fetal hemoglobin. They were found using the equilibrium constants of fetal hemoglobin in an analogous manner to the one described in [7] for the adult blood. These values are summarized in Table 3.
  Hemoglobin bounding with CO and O${}_{2}$ is cooperative, meaning that, once the first ligation happens, the following ones occurs more easily. Following that, a hemoglobin molecule is commonly unbounded or bounded with four other molecules. The exergy destruction rate is described as follow:
  (20) $${\dot{B}}_{d}={\dot{B}}_{ar,m}+{\dot{B}}_{ar,f}+{\dot{B}}_{M}-{\dot{B}}_{ve,m}-{\dot{B}}_{ve,f}-\dot{Q}\left(1-T_{0}/T_{c}\right)+\Delta {\dot{B}}_{blood,reaction}$$
  ΔB is given by:
  (21) $$\Delta B=\Delta {\dot{B}}_{O_{2_{maternal}}}+\Delta {\dot{B}}_{O_{2_{fetal}}}+\Delta {\dot{B}}_{CO_{maternal}}+\Delta {\dot{B}}_{CO_{fetal}}$$
- **Model 3**: Entropy generation caused by spontaneous diffusion.
  The entropy variation for each gas in the placenta is calculated by:
  (22) $$\Delta {\dot{S}}_{CO_{2}}=-\left({\dot{m}}_{CO_{2_{ve}}}-{\dot{m}}_{CO_{2_{ar}}}\right)R_{CO_{2}}\ln\frac{P_{CO_{2_{m,ve}}}}{P_{CO_{2_{f,ve}}}}$$
  (23) $$\Delta {\dot{S}}_{CO}=-\left({\dot{m}}_{CO_{ar}}-{\dot{m}}_{CO_{ve}}\right)R_{CO}\ln\frac{P_{CO_{f,ve}}}{P_{CO_{m,ve}}}$$
  (24) $$\Delta {\dot{S}}_{O_{2}}=-\left({\dot{m}}_{O_{2_{ar}}}-{\dot{m}}_{O_{2_{ve}}}\right)R_{O_{2}}\ln\frac{P_{O_{2_{f,ve}}}}{P_{O_{2_{m,ve}}}}$$
  In these equations, the pressure in each gas is in the maternal (m) and fetal (f) blood, although only the values from venous (ve) blood is used. Then, the entropy generation is given by Equation (25) and exergy destruction by Equation (26).
  (25) $$\dot{\sigma }=\Delta {\dot{S}}_{CO_{2}}+\Delta {\dot{S}}_{CO}+\Delta {\dot{S}}_{O_{2}}$$
  (26) $${\dot{B}}_{d}=T_{0}\dot{\sigma }$$

## 3. Results and Discussion

### 3.1. Maternal

**Figure 3:**
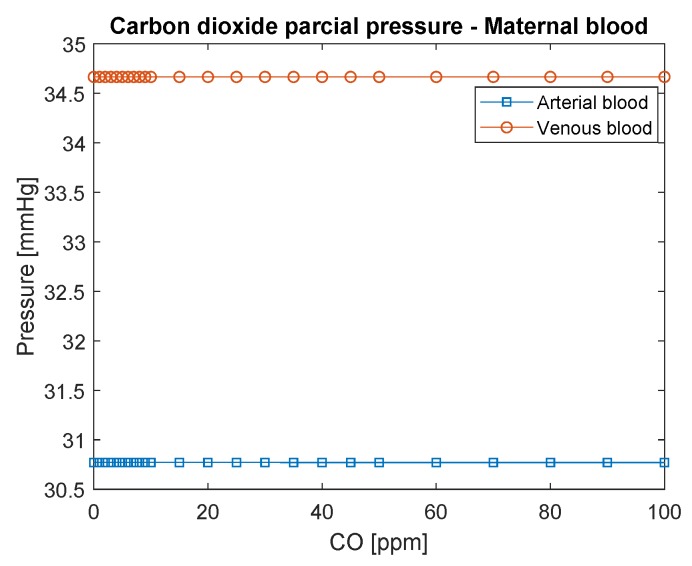
CO${}_{2}$ partial pressure in maternal blood. The conversion of CO in ppm to kg/m${}^{3}$ is to divide by 0.001.

Figure 3 shows the partial pressure of carbon dioxide (CO${}_{2}$) in maternal blood as a function of carbon monoxide concentration in the environment (CO-ppm). It is possible to observe that $CO_{2}$ does not change significantly with the increase of CO concentration, mainly because the mechanism of transport of $CO_{2}$ is different. Carbon dioxide pressure is lower than that found in the literature, possibly due to the increase in ventilation that occurs in pregnancy, as discussed by [25].

**Figure 4:**
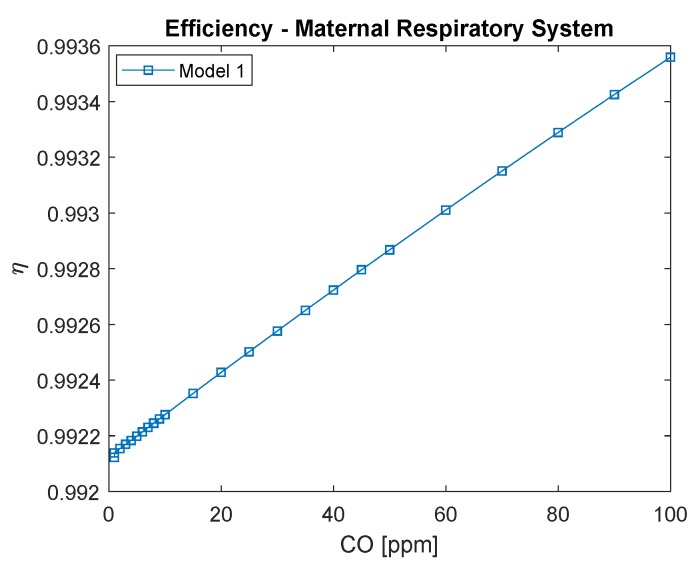
Maternal respiratory system exergy efficiency. The conversion of CO in ppm to kg/m${}^{3}$ is to divide by 0.001.

Maternal exergy efficiency is presented in Figure 4, bearing in mind that it can only be evaluated for Model 1, where it is possible to define the exergy of each stream, and therefore not only the change of exergy in hemoglobin bound as carried out in Model 2. It increases for CO poisoning, as found for a male in previous work [7], but with significantly higher efficiency, which can be associated with the enhanced ventilation undergone throughout the pregnancy.

**Figure 5:**
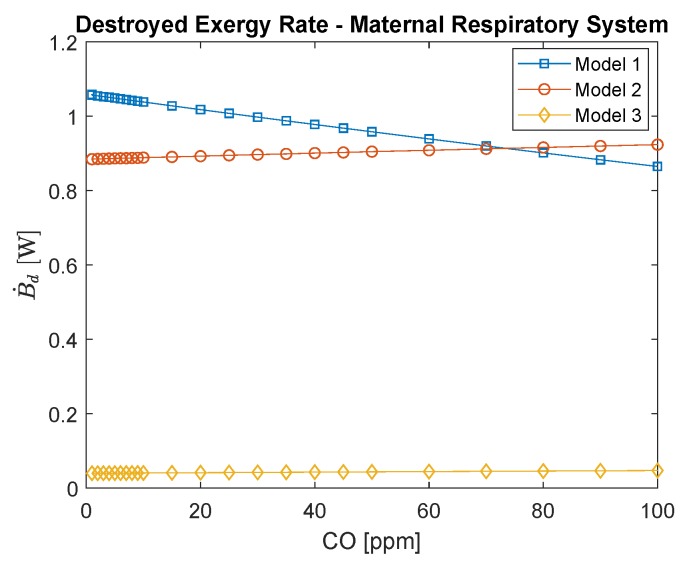
Maternal respiratory system destroyed exergy rate as a function of carbon monoxide intoxication in ppm. The conversion of CO in ppm to kg/m${}^{3}$ is to divide by 0.001.

Figure 5 shows for the maternal respiratory system the destroyed exergy rate evaluated by the three different models. Regarding the first method, the results show a decrease in the exergy destroyed rate with carbon monoxide poisoning (which may be misleading if related to the exergy efficiency that increases with the intoxication of CO), while for the other techniques this trend grows; higher mass transfer increases the irreversibilities. In the third method, the exergy destruction rate is the smallest among them, since metabolism and work are not considered, solely the diffusion and its irreversibilities. Nevertheless, it can be used to support the development of the second method, once they agree in their general behavior. Moreover, the fact that most of the irreversibilities of the lungs are not in the transport phenomena yet related to other terms, such as metabolic rate, may exhibit an evolutionary mechanism in decreasing this physical quantity.

**Figure 6:**
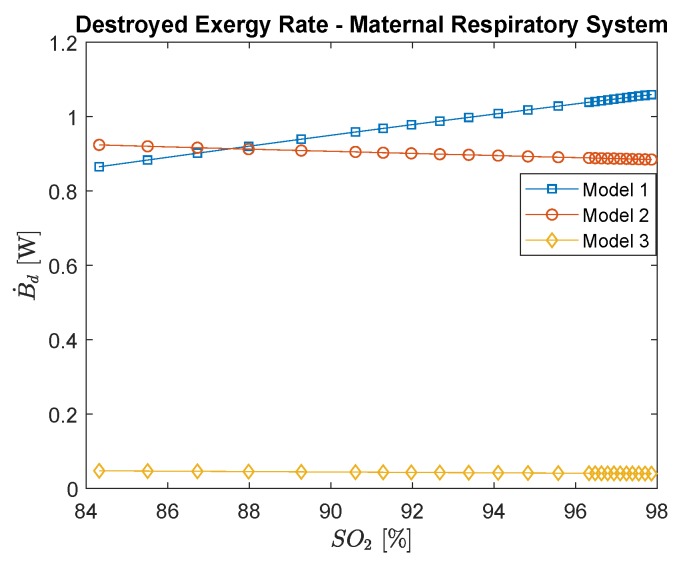
Maternal respiratory system destroyed exergy rate per oxyhemoglobin (saturation of O${}_{2}$ in the blood), which is a function of carbon monoxide poisoning.

Figure 6 shows the destroyed exergy rate per oxyhemoglobin percentage in arterial blood; comparing this graphic to Figure 5, it is possible to infer how blood saturation with O${}_{2}$ changes as CO poisoning increases. Moreover, the saturation of O${}_{2}$, or SO${}_{2}$, is one of the most applied indexes in medical practice to evaluate the gas exchanges in the lung (usually called lungs capacity) when the person is under a respiratory infection [20,24]. Therefore, lower values of SO${}_{2}$ indicate the necessity of a clinical intervention since the person is suffering from hypoxia. Usually, for a healthy subject, the saturation is higher than 96% [24]. Interestingly, the slope of the curves of the destroyed exergy is negative, from lower (such as 84%) to higher oxygen saturation figures (96–98%), which represents a healthy functioning of the lung (lower concentrations of CO in blood and environment).

### 3.2. Fetal Exergy Behavior

**Figure 7:**
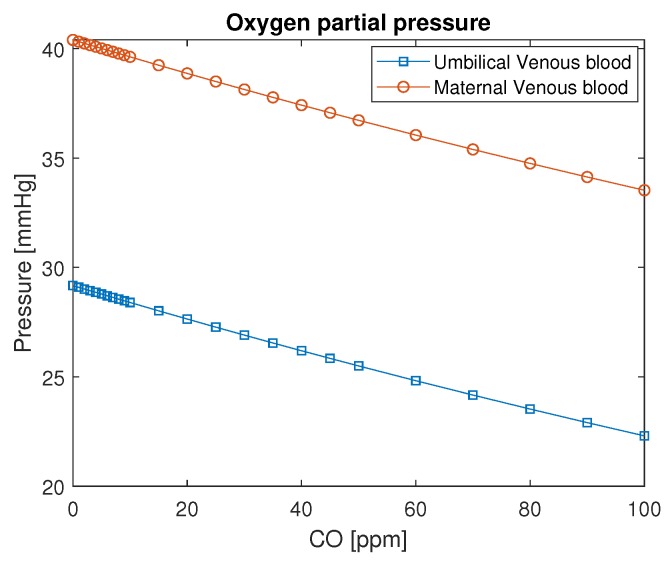
O${}_{2}$ partial pressure in maternal and fetal blood as a function of carbon monoxide intoxication in ppm. The conversion of CO in ppm to kg/m${}^{3}$ is to divide by 0.001.

**Figure 8:**
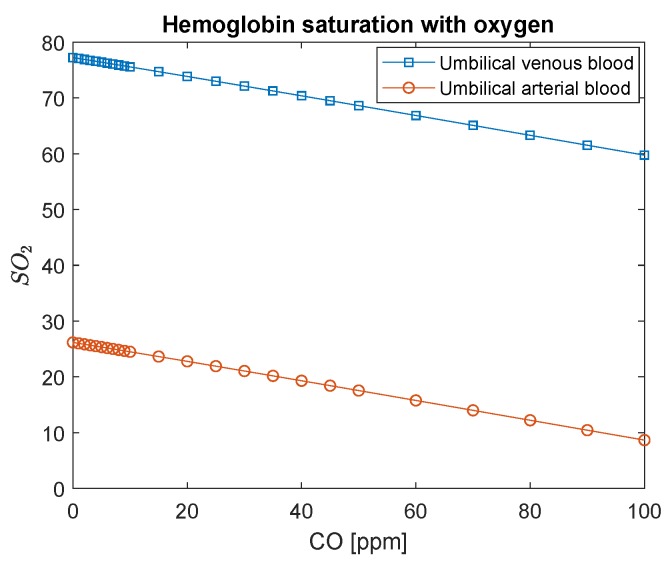
Fetal hemoglobin saturation with oxygen as a function of carbon monoxide intoxication in ppm. The conversion of CO in ppm to kg/m${}^{3}$ is to divide by 0.001.

The difference in pressure among maternal venous blood and fetal venous blood remains constant in this model (Figure 7) to ensure that oxygen diffuses through the placenta from the mother to the fetus. It is evident in this figure that, if the mother is intoxicated by values as high as 100 ppm, the partial pressure of O${}_{2}$ in the fetus’s blood will achieve considerably lower values: 20–25 mmHg. This condition may be seen in Figure 8. The saturation of oxygen in the fetus varies from values around 30 % to 10 %. This amount of oxygen may not be sufficient for the fetus’s metabolism, which may lead to hypoxia.

**Figure 9:**
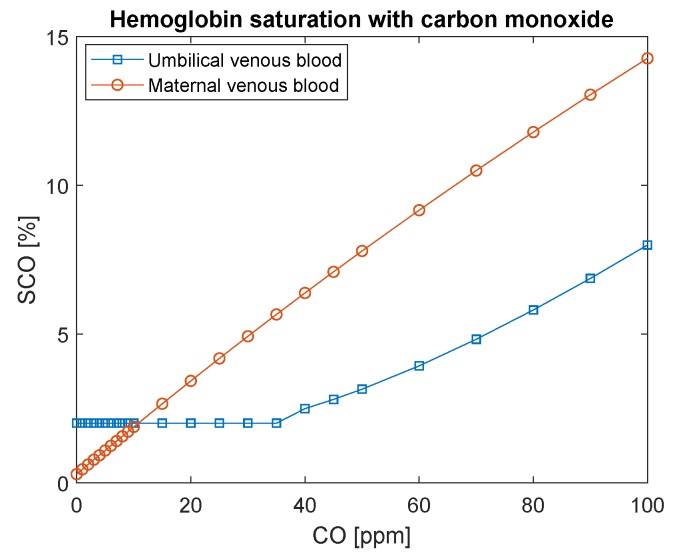
Hemoglobin saturation with carbon monoxide in maternal and fetal blood as a function of carbon monoxide intoxication in ppm. The conversion of CO in ppm to kg/m${}^{3}$ is to divide by 0.001.

On the other hand, Figure 8 indicates that hemoglobin saturation with O${}_{2}$ would need to decrease to significantly low values in fetal arterial blood to maintain the supply of oxygen constant (through fetal O${}_{2}$ gradient with the mothers). Recall that hemoglobin saturation with carbon monoxide in fetal blood never reaches maternal levels (Figure 9). It is assumed that its initial concentration is 2% [22] because, even when the mother is not exposed to CO, it is the usual concentration found in healthy fetus blood.

**Figure 10:**
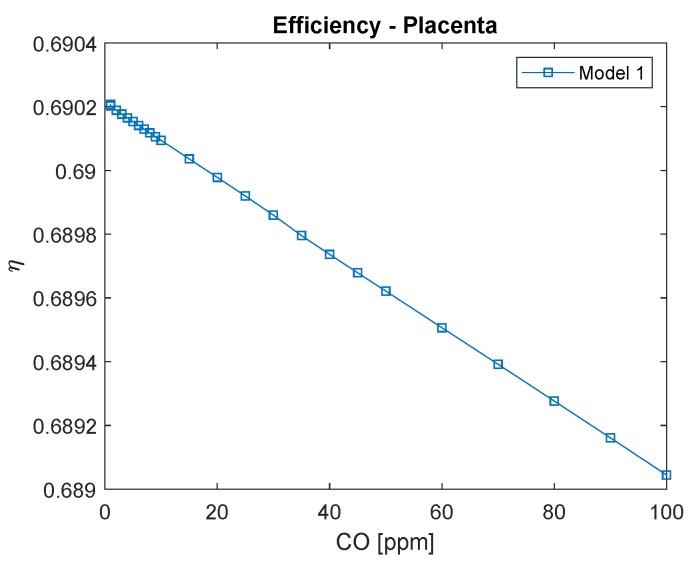
Placenta’s exergy efficiency as a function of carbon monoxide intoxication in ppm. The conversion of CO in ppm to kg/m${}^{3}$ is to divide by 0.001.

As shown in Figure 10 (Model 1), the placenta exergy efficiency is considerably low compared to the respiratory system. It may be explained by its high metabolism (which leads to high irreversibilities) and because it has functions other than providing oxygen to the fetus (which was not taken into consideration in the present analysis). This efficiency, calculated by Equation (18), is a function of the high ${\dot{B}}_{M}$, which becomes evident by the variation of this physical property as a function of CO in the air occur only in the third decimal place.

**Figure 11:**
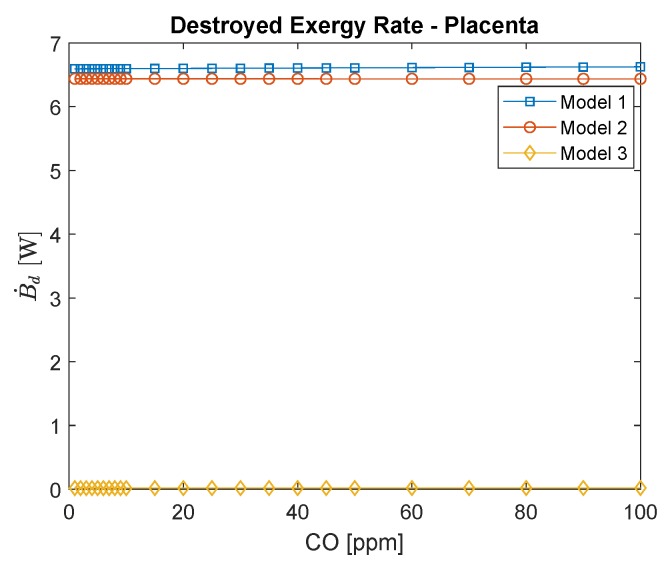
Placenta’s exergy destruction rate as a function of carbon monoxide intoxication in ppm. The conversion of CO in ppm to kg/m${}^{3}$ is to divide by 0.001.

Figure 11 shows the destroyed exergy rate for the placenta. The first and second analyses provide results that differ from the ones found for the respiratory system. The primary analysis results in a decrease in efficiency with CO poisoning and an increase in destroyed exergy rate, while the second analysis results in a reduction of the exergy destruction rate. Only the third analysis results in a rise in the destruction rate for both the respiratory system and the placenta. Nevertheless, this model results in destroyed exergy with an order of magnitude lower, indicating that, for the placenta, the percentages of irreversibilities associated with the mass transfer are not as high as they are for the lungs. This result is relevant because both organs are responsible for communications of the blood with an external environment (for the fetus and mother). This fact indicates the importance of the placenta, which is not only for mass transportation but also to sustain life. Nevertheless, it is vital to bear in mind that the irreversibilities as a function of carbon monoxide increases.

**Figure 12:**
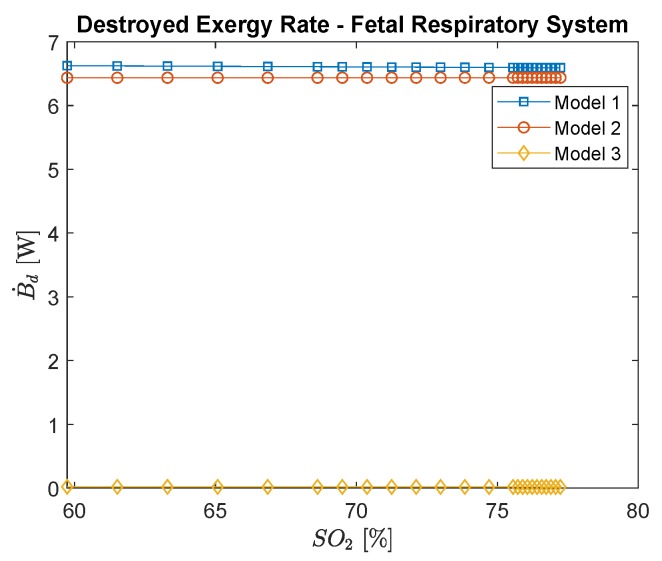
Fetal respiratory system destroyed exergy rate per oxyhemoglobin (saturation of oxygen), which is a function of carbon monoxide poisoning.

The graphic of the destroyed exergy as a function of the percentage of hemoglobin saturated with oxygen in the umbilical venous blood (Figure 12) is similar to Figure 11, because the destruction rate is mainly due to the metabolism, therefore they appear to be constant, which in fact is not a reality, since the intoxication of CO changes the irreversibilities of placenta, but with a lower order of magnitude.

## 4. Conclusions

In the past decade, the exergy analysis of biological systems has been a focus of attention, considering that the destroyed exergy and exergy efficiency are quality indexes in an energy conversion process. These are used to estimate indexes of thermal comfort [18], health care [13,14], and even for performance in sports [9,11,12]. In this study, the effect of carbon monoxide intoxication in a pregnant woman was analyzed.

The phenomenological model of the mother [20] and exergy models [6,7] were taken from literature. This article proposes a new phenomenological model of the placenta. Moreover, it has a distinguishing point, because it is the first attempt to use the respiratory model applied to the mother and fetus in energy and exergy basis.

In the analyses, the destroyed exergy rate for three models was calculated. The first model took into account the gases dissolved in the blood only as ideals. The second method associated the Gibbs free energy variation of the hemoglobin reaction with CO and O${}_{2}$. Finally, the third model considered only irreversibilities related to gas transportation.

Regarding the mother’s exergy behavior of the lungs, for the first model, a decrease in the destroyed exergy as a function of the intoxication of CO was obtained. The second and third models had similar trends, where the destroyed exergy increases as a function of the CO poisoning. Moreover, the third model indicates the irreversibilities of the mass transfer, which corroborates the results of the second model.

It was also possible to conclude that the main effects in the fetus are caused by hypoxia due to the low oxygen pressures in the fetus’s blood. In this analysis, the HbCO concentration was found to be smaller in the fetus’s blood than in the mothers. The exergy efficiency of the maternal respiratory system, calculated from Model 1, is higher than the average male. This can be related to increased ventilation of the lungs during pregnancy. Moreover, Models 2 and 3 indicated a similar trend, which suggests that the main modifications in the lung are associated with the increase of irreversibilities of mass transfer.

The placenta destroyed exergy rate is significantly higher than the maternal, due to its high metabolism. The third analysis is useful in this case because it shows that the exergy destroyed rate increases, although to a small amount. This is the reason that makes the other two analyses appear without a slope.

Eventually, the destroyed exergy rate was correlated with an indicator often used in the medical area, which is the saturation of oxygen in the hemoglobin, showing that the exergy analysis may be a proper index to evaluate the human body in healthy and unhealthy scenarios. This type of study may be incorporated in the medical area first because the exergy analysis may give a complementary diagnosis of diseases. Second, with the phenomenological model, it is possible to suggest different scenarios of intoxication without the necessity of experiments in living beings. A limitation of this study is regarding the saturation curve of the mother subjected to some poisoning, which suffers a change; moreover, the two phenomenological models could be integrated to obtain a more extensive range of results of a pregnant woman.

## Figures and Tables

**Table 1:** Parameters obtained in the literature representing the pregnant woman to change some physiological settings of the model proposed by Albuquerque Neto et al. [20].

Parameter	Value	Reference
Cardiac Output	7.2 L·min${}^{-1}$	[25]
Blood Volume	6.53 L	[26]
Oxygen Consumption	249 mL·min${}^{-1}$	[26]
Ventilation	8.51 L·min${}^{-1}$	[26]
Hemoglobin Concentration	12.1 g·dL${}^{-1}$	[26]
Respiratory Quotient	0.83	[26]

**Table 2:** Parameters of umbilical and fetal blood flow obtained in literature.

Parameter	Value	Reference
Umbilical blood flow	66 mL·min${}^{-1}$·kg${}^{-1}$	[28]
Fetal O${}_{2}$	6.53 L	[28]
Hemoglobin	14.5 g·dL${}^{-1}$	[28]
PO${}_{2}$ venous umbilical blood	28.8 mmHg	[28]

**Table 3:** Gibbs free energy for fetal hemoglobin in (kJ/mol). Based on systematization proposed by Cenzi et al. [7] for biological reference conditions.

	$\Delta_{f}G^{{}^{\prime }o}{\left(\mathrm{Hb}\left(\mathrm{CO}\right)\right)}_{4}$	$\Delta_{f}G^{{}^{\prime }o}{\left(\mathrm{Hb}\left(O_{2}\right)\right)}_{4}$
$\Delta_{f}G^{{}^{\prime }o}\left(Hb\left(E\right)\right)$	−150.09	−18.34
$\Delta_{f}G^{{}^{\prime }o}{\left(Hb\left(E\right)\right)}_{2}$	−301.04	−37.41
$\Delta_{f}G^{{}^{\prime }o}{\left(Hb\left(E\right)\right)}_{3}$	−464.96	−59.86
$\Delta_{f}G^{{}^{\prime }o}{\left(Hb\left(E\right)\right)}_{4}$	−634.09	−95.89

## Abbreviations

The following abbreviations and symbols are used in this manuscript:

**Symbols**	
**B**	Exergy of the control volume (J)
$\dot{B}$	Exergy rate (W)
*b*	Specific exergy (kJ/kg)
*C*	Concentration (kg/m${}^{3}$)
*D*	Diffusion coefficient (mL/(kg.min))
*G*	Gibbs free energy (kJ)
*g*	specific gibbs energy (kJ/kg)
$\dot{H}$	Enthalpy rate (W)
*h*	Specific enthalpy (kJ/kg)
$\dot{m}$	Mass flow rate (kg/s)
$\dot{M}$	Energy metabolism (W)
*P*	Pressure (kPa, mmHg)
$\dot{Q}$	Heat transfer rate (W)
*R*	Gas constant (kJ/kgK)
RQ	respiratory quotient (-)
$\dot{S}$	Entropy rate (W/K)
*s*	Specific entropy (kJ/kgK)
*T*	Temperature (${}^{\circ }$C)
*t*	Time (s)
$\dot{V}$	Volumetric flow rate (m${}^{3}$/s)
*V*	Volume (m${}^{3}$)
*v*	Specific volume (m${}^{3}$/kg)
$\dot{W}$	Power output (W)

**Greek letters**	
α	solubility coefficient of the gas in the blood (ml${}_{O_{2}}$)
η	efficiency (-)
ρ	specific mass (kg/m${}^{3}$)
$\dot{\sigma }$	entropy generation rate (kW/K)

**subscripts**	
0	reference state
ar	arterial blood
air	environment air
blood	blood
CV	control volume
*d*	destroyed
diffusion	Diffusion
*e*	exit
exp	expired air
*f*	fetus
Hb	reacted with hemoglobin
*i*	inlet
lung	lung
*m*	mother
*M*	associated with the metabolic heat
ve	venous blood
